# Social and structural drivers of HIV vulnerability among a respondent‐driven sample of feminine and non‐feminine presenting transgender women who have sex with men in Zimbabwe

**DOI:** 10.1002/jia2.26231

**Published:** 2024-04-16

**Authors:** Lauren E. Parmley, Sophia S. Miller, Innocent Chingombe, Munyaradzi Mapingure, Owen Mugurungi, John H. Rogers, Godfrey Musuka, Chesterfield Samba, Avi J. Hakim, Tiffany G. Harris

**Affiliations:** ^1^ ICAP at Columbia University New York New York USA; ^2^ ICAP at Columbia University Harare Zimbabwe; ^3^ Zimbabwe Ministry of Health and Child Care Harare Zimbabwe; ^4^ Division of Global HIV & TB U.S. Centers for Disease Control Harare Zimbabwe; ^5^ Gay and Lesbians of Zimbabwe Harare Zimbabwe; ^6^ Division of Global HIV & TB U.S. Centers for Disease Control Atlanta Georgia USA; ^7^ Department of Epidemiology Columbia University Mailman School of Public Health New York New York USA

**Keywords:** transgender people, key and vulnerable populations, human rights, Zimbabwe, structural drivers, Africa

## Abstract

**Introduction:**

We sought to characterize social and structural drivers of HIV vulnerability for transgender women (TGW) in Zimbabwe, where TGW are not legally recognized, and explore differences in vulnerability by feminine presentation.

**Methods:**

A secondary analysis was conducted with a sub‐sample of participants recruited from a 2019 respondent‐driven sampling survey that comprised men who have sex with men, TGW and genderqueer individuals assigned male sex at birth, from two cities in Zimbabwe. Survey questionnaires captured information related to socio‐demographics, sexual and substance use behaviours, and social and structural barriers to HIV services. Secondary analyses were restricted to participants who identified as female, transfemale or transwomen (236/1538) and were unweighted. Descriptive statistics were used to calculate sample estimates and chi‐square and Fisher's exact tests were used to assess differences in vulnerability by feminine presentation.

**Results:**

Among 236 TGW, almost half (45.3%) presented as feminine in the 6 months preceding the survey and 8.5% had ever used hormones to affirm their gender identities. Median age among TGW was 23 years (interquartile range: 20–26). Feminine presenting TGW in our sample had higher prevalence of arrest (15.9% vs. 3.9%), rejection by family/friends (38.3% vs. 14.0%), employment termination (11.2% vs. 3.9%), employment refusal (14.0% vs. 3.9%), denial of healthcare (16.8% vs. 2.3%), physical, sexual or verbal harassment or abuse (59.8% vs. 34.1%), alcohol dependence (32.7% vs. 12.4%), recent transactional sex with a male or TGW partner (30.8% vs. 13.3%) and recent non‐injection drug use (38.3% vs. 20.2%) than non‐feminine presenting TGW (all *p*‐value <0.05).

**Conclusions:**

Findings suggest that TGW, particularly feminine presenting TGW, experience social and structural inequities which may contribute to HIV vulnerability. Interventions aimed at addressing inequities, including trans competency training for providers and gender‐affirming, psychosocial and legal support services for TGW, might mitigate risk.

## 1 INTRODUCTION

Transgender people, including transgender women (TGW), are disproportionately affected by HIV [1, 2, 3] and face distinct challenges accessing HIV prevention and treatment services. In 2021, the relative risk of acquiring HIV was 14 times higher among TGW than adult women in the general population globally [4]. In the same year, 2% of all new HIV acquisitions were estimated to occur among TGW [4].

While available estimates on the burden of HIV among TGW reveal health inequities across contexts, estimates on HIV‐related vulnerabilities and the continuum of HIV services for TGW continue to be hampered by limited availability and quality of data, undermining efforts of a data‐driven response. For the 2017−2021 period, only 11 countries reporting on global AIDS monitoring indicators related to a country's HIV response for key populations (KP) had available data on recent avoidance of healthcare seeking by transgender people, seven had available data on recent physical and/or sexual violence and four had available data on recent experiences of stigma and discrimination [4].

HIV vulnerabilities, such as stigma, discrimination and gender‐based violence, are perpetuated by the criminalization of KP, including transgender, gender‐diverse people and men who have sex with men (MSM) [5]. Among the UNAIDS surveyed countries in 2022, at least 20 had legal and policy environments that formally criminalized and/or prosecuted transgender people (45 countries lacked data) and 70 that criminalized same‐sex sexual relations [4]. Pathways by which the legal and policy environment influence HIV outcomes have been explored for some KP [6, 7, 8, 9, 10], and may include both enacted and perceived stigma.

In Zimbabwe, legal protections for KP, including MSM and transgender persons, are non‐existent. Trans or non‐binary/genderqueer persons are not legally recognized and there is no provision for transgender people to update their gender on identification documents or official records [11]. Same‐sex sexual relationships are criminalized in Zimbabwe, and without legal recognition, TGW who have sex with men risk detention [11]. Access to gender‐affirming care, including hormone therapy and gender affirmation surgery, is absent, and there are documented reports of transgender people accessing hormone therapy through the black‐market or by travelling to South Africa [12, 13]. Hormone therapy in the absence of a medical provider poses health risks, and international travel to access routine health services has cost implications, further compounding vulnerabilities for TGW who access gender‐affirming care through what may be their only means.

We sought to document the social and structural drivers of HIV vulnerability for TGW in Zimbabwe and explore differences by feminine presentation as part of a secondary data analysis. We hypothesized that feminine presenting TGW may experience greater vulnerabilities than non‐feminine presenting TGW considering the social and legal context in Zimbabwe. Findings from this analysis contribute towards the evidence base on TGW in sub‐Saharan Africa, an underrepresented group in HIV research, and could inform a more trans‐inclusive HIV response in the country.

## 2 METHODS

### 2.1 Study design

Survey methods and primary outcomes have been published elsewhere [14, 15]. Briefly, individuals in Harare and Bulawayo, Zimbabwe were recruited to participate in a cross‐sectional biobehavioural survey using respondent‐driven sampling (RDS), a peer‐chain referral approach designed to recruit populations for whom no sampling frame exists [16], from March to July 2019. Individuals were eligible to participate if they were male sex assigned at birth (irrespective of gender identity), had anal/oral sex with a man in the past 12 months, ≥18 years, resided in the city for ≥1 month and spoke English/Shona/Ndebele. Nineteen purposively selected seeds (Harare: 11; Bulawayo: 8), four of which identified as TGW, were provided three coupons and encouraged to recruit their peers; those recruited also received three coupons and the process continued until the target sample size was reached (Harare: 718; Bulawayo: 820).

### 2.2 Procedures

Survey procedures were aligned to those outlined in the World Health Organization Biobehavioral Survey Guidelines [17]. Individuals were screened for eligibility and if eligible and interested, provided written informed consent. A tablet‐based questionnaire was administered to participants. Interview domains included demographic characteristics, sexual orientation and gender identity, HIV risk behaviours, social and structural vulnerabilities, including experiences of stigma and discrimination, and health‐seeking behaviours. Consenting participants were tested for HIV, syphilis, and hepatitis B and were referred for care if the results were positive. All participants were reimbursed a maximum of US$25 to cover transport costs and time.

### 2.3 Ethics

Ethical approvals were received from the Columbia University Institutional Review Board and the Medical Research Council of Zimbabwe. The protocol was also reviewed per CDC human research protection procedures and was determined to be research, but CDC investigators did not interact with human subjects or have access to identifiable data or specimens for research purposes.

### 2.4 Measures

Gender identity was assessed using a two‐step question. Participants were first asked their current gender followed by their sex assigned at birth. Gender response options were male, female, transmale/transman, transfemale/transwoman, genderqueer (a non‐binary term used in Zimbabwe) and other which, if selected, was followed by a free‐text response. The analytic sample was restricted to participants who identified their current gender as female, transfemale or transwoman, hereafter referred to as TGW. All participants who identified as TGW received an additional series of questions related to their gender identity and experiences. For example, participants were asked “In the past 6 months, have you ever lived as a woman? By living as a woman, I mean dressing and presenting yourself as a woman.” Those who answered “yes” were categorized to be feminine presenting. Validated measures in the questionnaire included the Alcohol Use Disorders Identification Test (AUDIT) and the Patient Health Questionnaire‐2 (PHQ‐2) [18, 19]. Alcohol dependence was assessed using an AUDIT score ≥15 [18] and major depressive disorder was assessed using a PHQ‐2 score ≥3 [19]. Transactional sex was defined as having received money, goods or services for sex with one or more male or TGW partners in the past 6 months.

### 2.5 Data analysis

Descriptive statistics were used to calculate sample prevalence estimates and chi‐square and Fisher's exact tests (when expected cell counts <5) were used to assess differences in prevalence among TGW by feminine presentation. All analysis was conducted in SAS 9.4 (Cary, NC). Data were treated as a convenience sample and analysis did not account for sampling design as the original RDS sample did not reach convergence on key variables aligned with primary study objectives, including HIV.

## 3 RESULTS

### 3.1 Sample characteristics and participant demographics

Among the original sample, 77.6% (1194/1538) identified as male, 8.8% (135/1538) identified as female, 6.6% (101/1538) identified as transfemale/transwoman and 7.0% (108/1538) identified as genderqueer/non‐binary. Among TGW in the analytic sample (*N* = 236), most were single (91.5%) and identified as gay/homosexual (86.4%) (Table 1). Median age among TGW was 23 years (interquartile range [IQR]: 20–26). TGW most reported disclosing their gender identity to friends who were gay/lesbian (Table 1). Almost half (45.3%) of TGW dressed/presented as feminine in the 6 months preceding the survey and 8.5% had ever used hormones to affirm their gender identities. Feminine presentation was more common among TGW in Bulawayo compared to Harare (86.8% vs. 37.4%; *p*‐value <0.0001) (Table 1).

**Table 1:** Demographic and gender‐related characteristics among a sample of feminine and non‐feminine presenting TGW in Harare and Bulawayo, Zimbabwe (*N* = 236), 2019

	Total (*N* = 236)		Non‐feminine presenting TGW (*N* = 129)		Feminine presenting TGW (*N* = 107)		
Characteristic	col%	*n*/*N*	row%	*n*/*N*	row%	*n*/*N*	χ^2^ or Fisher's exact tests *p*‐value
Age, median (IQR)	23 (20−26)		22 (20−24)		23 (20−28)		
City							<0.0001
Bulawayo	16.1	(38/236)	13.2	(5/129)	86.8	(33/107)	
Harare	83.9	(198/236)	62.6	(124/129)	37.4	(74/107)	
Marital status							1.0
Single, never married	91.5	(216/236)	54.6	(118/129)	45.4	(98/107)	
Married or cohabitating	1.7	(4/236)	50.0	(2/129)	50.0	(2/107)	
Separated/divorced or widowed	6.8	(16/236)	56.3	(9/129)	43.8	(7/107)	
Highest education attended							0.9664
None or Primary	3.4	(8/236)	50.0	(4/129)	50.0	(4/107)	
Secondary	74.2	(175/236)	54.3	(95/129)	45.7	(80/107)	
Tertiary or Vocational	22.5	(53/236)	56.6	(30/129)	43.4	(23/107)	
Nationality							0.0918
Zimbabwean	98.7	(233/236)	55.4	(129/129)	44.6	(104/107)	
Other African	1.27	(3/236)	0	(0/129)	100.0	(3/107)	
Sexual orientation							0.0066
Gay/homosexual	86.4	(204/236)	52.0	(106/129)	48.0	(98/107)	
Bisexual	12.7	(30/236)	76.7	(23/129)	23.3	(7/107)	
Straight/heterosexual	0	(0/236)	0	(0/129)	0	(0/107)	
Other	0.9	(2/236)	0	(0/129)	100.0	(2/107)	
Disclosed gender identity to^a^							
Transgender friends	72.5	(171/236)	46.8	(80/129)	53.2	(91/1.7)	<0.0001
Gay/Lesbian friends who are not transgender	95.8	(226/236)	54.4	(123/129)	45.6	(103/107)	1.0
Heterosexual friends who are not transgender	39.0	(92/236)	39.1	(36/129)	60.9	(56/107)	0.0001
Family	35.6	(84/236)	35.7	(30/129)	64.3	(54/107)	<0.0001
Spouse	3.4	(8/236)	25.0	(2/129)	75.0	(6/107)	0.1456
Healthcare provider	18.2	(43/236)	25.6	(11/129)	74.4	(32/107)	<0.0001
Ever used hormones to affirm gender identity							0.0011
No	91.5	(216/236)	57.9	(125/129)	42.1	(91/107)	
Yes	8.5	(20/263)	20.0	(4/129)	80.0	(16/107)	

Abbreviations: IQR, interquartile range; TGW, transgender women.

^a^ Responses not mutually exclusive.

### 3.2 Social and structural drivers of HIV vulnerability

Nearly half (45.8%) of TGW reported experiencing physical, sexual or verbal abuse because they have sex with men (Table 2). Of those, 71.3% had experienced abuse within 6 months preceding the survey. Abuse was perpetrated by friends (58.3%), strangers (50.9%), family members (21.3%), sex partners (12.0%), uniformed services personnel (9.3%), authority figures (8.3%) and healthcare workers (7.4%). Approximately 1 in 10 TGW had ever been arrested because they have sex with men (9.3%), 1 in 5 had alcohol dependence (21.6%) and 1 in 3 were unemployed (38.6%).

**Table 2:** Associations of feminine presentation and social and structural drivers of HIV among a sample of TGW in Harare and Bulawayo, Zimbabwe, 2019

	Total (*N* = 236)		Non‐feminine presenting TGW (*N* = 129)	Feminine presenting TGW (*N* = 107)	χ2 or Fisher's exact test *p*‐value
	%	*n*/*N*	%	%	
**Arrest because you have sex with men**	**9.3**	**(22/236)**	**3.9**	**15.9**	**0.0016**
**Rejection by family/friends because you have sex with men**	**25.0**	**(59/236)**	**14.0**	**38.3**	**<0.0001**
Unemployment	38.6	(91/236)	41.9	34.6	0.2526
**Employment termination because you have sex with men**	**7.2**	**(17/236)**	**3.9**	**11.2**	**0.0299**
**Employment refusal because you have sex with men**	**8.5**	**(20/236)**	**3.9**	**14.0**	**0.0053**
**Blackmail because you have sex with men**	**17.8**	**(42/236)**	**12.4**	**24.3**	**0.0174**
Avoidance of seeking healthcare because you were worried someone may learn you have sex with men	22.9	(54/236)	18.6	28.0	0.0859
**Unfair treatment by healthcare provider or denial of healthcare because you have sex with men**	**8.9**	**(21/236)**	**2.3**	**16.8**	**<0.0001**
**Physical, sexual, or verbal harassment or abuse because you have sex with men**	**45.8**	**(108/236)**	**34.1**	**59.8**	**<0.0001**
**Alcohol dependence** ^a^	**21.6**	**(51/236)**	**12.4**	**32.7**	**0.0002**
**Non‐injection drug use in the past 6 months**	**28.4**	**(67/236)**	**20.2**	**38.3**	**0.0021**
**Transactional sex with a male or TGW partner in the past 6 months** ^**b**^	**21.3**	**(50/235)**	**13.3**	**30.8**	**0.0011**
Major depressive disorder^c^	16.1	(38/236)	15.5	16.8	0.7838

*Note*: Boldface font denotes statistical significance at *p*‐value <0.05.

Abbreviations: AUDIT, Alcohol Use Identification Test; PHQ, Patient Health Questionnaire; TGW, transgender women.

^a^ AUDIT score of ≥15.

^b^ *n* = 1 missing.

^c^ PHQ‐2 score ≥3.

Feminine presenting TGW had higher prevalence of arrest (15.9% vs. 3.9%), rejection by family/friends (38.3% vs. 14.0%), employment termination (11.2% vs. 3.9%), employment refusal (14.0% vs. 3.9%), blackmail (24.3% vs. 12.4%), unfair treatment by healthcare provider or denial of healthcare (16.8% vs. 2.3%) and physical, sexual or verbal harassment or abuse (59.8% vs. 34.1%) because they have sex with men, alcohol dependence (32.7% vs. 12.4%), recent transactional sex with a male or TGW partner (30.8% vs. 13.3%) and recent non‐injection drug use (38.3% vs. 20.2%) than non‐feminine presenting TGW (all *p*‐value <0.05) (Table 2). There was no difference in the levels of major depressive disorder (16.8% vs. 15.5%), unemployment (34.6% vs. 41.9%) or avoidance of seeking healthcare out of worry someone may learn they have sex with men (28.0% vs. 18.6%) between feminine and non‐feminine presenting TGW (all *p*‐value ≥0.05).

## 4 DISCUSSION

Advancing HIV and health services for TGW populations in sub‐Saharan Africa necessitates improved data on and understanding of TGW. Taken together, findings support our hypothesis that feminine presenting TGW may experience greater vulnerabilities than non‐feminine presenting TGW and contribute to the limited body of evidence on HIV‐related research among this group.

Consistent with findings in the region [20], data suggest that TGW are highly vulnerable to social and structural vulnerabilities that contribute to health inequities. In a multi‐country analysis of TGW in sub‐Saharan Africa, TGW reported high levels of stigma and violence [20]; nearly 8% reported prior arrest, 30% reported rejection by friends as a result of sexual orientation or practice, 27% reported fear of seeking healthcare services due to sexual orientation or practice and 2% reported denial of healthcare services [20]. While our sample overall had comparable levels to those reported in this aggregate analysis, feminine presenting TGW in our study had higher levels of social and structural vulnerabilities compared to non‐feminine presenting TGW, demonstrating the importance of more nuanced approaches to understand risk vulnerabilities among TGW and suggesting that the presentation of TGW's gender may influence vulnerability profiles in non‐protective legal contexts. Interventions aimed at addressing social and structural factors that contribute to HIV and other health‐related vulnerabilities may mitigate these challenges and are important enablers to reaching the UNAIDS 10‐10‐10 targets to remove social and legal impediments to access and utilize HIV services [21].

In many contexts, gender‐affirming services, including trans‐competent providers and availability of hormone therapy, can support access to HIV prevention, care, and treatment services [22] and improved outcomes [23, 24, 25]. Equally, unmet need for gender affirmation may inhibit HIV‐related outcomes for TGW [26]. As earlier noted, hormone therapy was not available through the public health sector in Zimbabwe at the time of the survey and, therefore, may have been accessed through illegal and potentially unsafe means or accessed outside of Zimbabwe for participants using hormone therapy. Feminine presentation by participants may also, therefore, be an important indication of minimum interest in or demand for hormone therapy if it were made available in this context. However, additional data to better understand access to, use of and demand for hormone therapy among TGW in Zimbabwe are warranted to enable trans‐inclusive services. Moreover, the reported high prevalence of unfair treatment or denial of healthcare by providers in our sample underscores an urgent need for gender‐affirming services and provider sensitization on trans people in Zimbabwe, including the provision of psychosocial and legal support for TGW who, as demonstrated in our sample, experience high levels of stigma and discrimination and substance use.

These data represent a convenience sample and do not necessarily represent TGW in Harare and Bulawayo. While the small sample size limited our ability to run a multivariable model adjusting for potential confounders, such as city, distribution of vulnerabilities by feminine presentation was consistent when we restricted analyses to TGW in Harare alone (unpublished) with the exception of alcohol dependence, blackmail and employment refusal, suggesting the relationship between feminine presentation and increased experiences of most social and structural vulnerabilities may be similar in both cities. There are limitations of the feminine presentation measure used in this study, including that participants may have different definitions for living as a woman and that feminine presentation may change over time. One limitation of the primary study is that TGW were not explicitly recruited in the survey's formative assessment, and it was only after the formative assessment was conducted that stakeholder feedback highlighted the need for TGW's inclusion in the study [27]. As a result, conflation of sexual orientation and gender identity terms, and understanding of gender identity in this context was not explored in‐depth. In our analysis, most TGW reported their sexual orientation as gay/homosexual which may appear to be surprising or incongruent with western understanding of gender identity. Further exploration on terminology and understanding of gender identity in Zimbabwe may elucidate these findings. Despite limitations, this is one of few HIV surveys which describe gender minorities in sub‐Saharan Africa. Findings warrant further study into the relationship between feminine presentation and HIV vulnerabilities for TGW in other contexts.

## 5 CONCLUSIONS

Findings suggest that TGW, particularly feminine presenting TGW, experience social and structural inequities, including stigma, discrimination, depression and substance abuse, which may contribute to HIV vulnerability in this context. Social and structural interventions, including trans competency training for providers and implementation of gender‐affirming, psychosocial and legal support services for TGW, to support an enabling, human rights‐affirming environment may alleviate these inequities.

## COMPETING INTERESTS

The authors have no competing interests to declare.

## AUTHORS’ CONTRIBUTIONS

LEP, SSM, IC, MM, OM, JHR, GM, CS, AJH and TGH contributed to designing the survey, developing the data collection instruments and implementing survey procedures. LEP wrote the initial manuscript draft and led the analysis. SSM contributed to the drafting of the manuscript and verified the underlying data. All authors critically reviewed the manuscript, approved the final manuscript and had final responsibility for the decision to submit for publication.

## FUNDING

This project was supported by the U.S. President's Emergency Plan for AIDS Relief through the CDC under the terms of cooperative agreement #U2GGH001939.

## DISCLAIMER

Lauren Parmley was not at USAID when the research for the current paper was conducted. The findings and conclusions in this manuscript are those of the authors and do not necessarily represent the official position of the funding agencies.

## Data Availability

The data that support the findings of this study are available on request from the corresponding author. The data are not publicly available due to privacy or ethical restrictions.
